# Endothelium-derived 6-nitrodopamine is the major mechanism by which nitric oxide relaxes the rabbit isolated aorta

**DOI:** 10.3389/fphar.2024.1507802

**Published:** 2024-11-21

**Authors:** Eric Xavier Dos Santos, José Britto-Júnior, João Victor Ribeiro, Gilberto Quirino Junior, Antonio Tiago Lima, Manoel Odorico Moraes, Maria Elisabete A. Moraes, Edson Antunes, André Schenka, Gilberto De Nucci

**Affiliations:** ^1^ Department of Pharmacology, Faculty of Medical Sciences, State University of Campinas (UNICAMP), Campinas, São Paulo, Brazil; ^2^ Department of Pharmacology, Faculty São Leopoldo Mandic, Campinas, São Paulo, Brazil; ^3^ Clinical Pharmacology Unit, Drug Research and Development Center, Federal University of Ceará (UFC), Fortaleza, Brazil; ^4^ Department of Pharmacology, Institute of Biomedical Sciences, University of São Paulo (USP), São Paulo, Brazil

**Keywords:** indomethacin, cyclo-oxygenase, cyclic GMP, acetylcholine, endothelium-dependent hyperpolarization factor

## Abstract

6-Nitrodopamine (6-ND) is the predominant catecholamine released from isolated vascular tissues in both mammals and reptiles, with its release being significantly reduced by the NO synthesis inhibitor, N^ω^-nitro-L-arginine methyl ester (L-NAME). The vasorelaxation induced by 6-ND is unaffected by either L-NAME or the soluble guanylate cyclase (sGC) inhibitor, ODQ, indicating an alternative mechanism of action. The vasorelaxant effect appears to be mediated through selective antagonism of dopamine D_2_ receptors rather than traditional nitric oxide (NO)-mediated pathways. This study examined the basal release of 6-ND, dopamine, noradrenaline, and adrenaline from the rabbit thoracic aorta by liquid chromatography coupled to tandem mass spectrometry (LC-MS/MS). Additionally, the effects of 6-ND and the dopamine receptor antagonist L741,626 on relaxation responses and electric-field stimulation (EFS)-induced contractions in aortic rings were assessed. Nitric oxide pathway inhibitors, including L-NAME, ODQ, and methylene blue, were utilized to assess the involvement of this pathway in 6-ND-induced vasorelaxation. Concentration–response curves for norepinephrine, epinephrine, and dopamine were generated in the presence and absence of 6-ND and L-741,626. The rabbit isolated aorta presented the basal release of endothelium-derived dopamine and 6-ND. Furthermore, 6-nitrodopamine and L-741,626 induced concentration-dependent relaxations in endothelin-1 pre-contracted aortic rings. The relaxations were reduced by the mechanical removal of the endothelium but unaffected by pre-treatment with L-NAME, ODQ, or methylene blue. Pre-incubation with 6-ND significantly reduced dopamine-induced contractions, while noradrenaline- and adrenaline-induced contractions remained unchanged. The findings demonstrated that endothelium-derived 6-ND is the most potent endogenous relaxant of the rabbit isolated aorta, and the mechanism is independent of the NO pathway and involved the blockade of dopamine D2 receptors.

## Introduction

The rabbit isolated aorta (Furchgott and Bhadrakom, 1953) is a classical tissue to study the release and action of vasoactive substances, such as nitric oxide (Furchgott and Zawadzki, 1980). Nitric oxide is reported to cause vascular smooth muscle relaxation by stimulating soluble guanylate cyclase (Katsuki et al., 1977). However, in rabbit aortic rings, the selective soluble guanylate cyclase (sGC) inhibitor NS 2028 (Olesen et al., 1998) almost abolishes the sodium nitroprusside (SNP)-induced cGMP increase, but it only partially inhibits the NO-mediated relaxant response to acetylcholine (Fleming et al., 1999), indicating that there is a substantial cGMP-independent component of the NO-mediated dilation in this tissue. Indeed, the increase in tonus induced by NS 2028 in the PGF_2α_-contracted aortic rings was markedly smaller than that induced by N^G^-nitro-L-arginine (Olesen et al., 1998).

6-Nitrodopamine (6-ND) is the major catecholamine released from isolated vascular tissues from mammalian and reptilian species, and its release/synthesis is significantly reduced when tissues are pre-incubated with the NO synthesis inhibitor N^ω^-nitro-L-arginine methyl ester (L-NAME; Zatz and De Nucci, 2024). The balance between endothelium-derived dopamine and 6-ND has been proposed as the major mechanism for controlling vascular smooth-muscle (Britto-Júnior et al., 2021; Britto-Júnior et al., 2023) and corpus cavernosum contractility (Lima et al., 2023). In reptilian aortic rings (Lima et al., 2022), human umbilical cord vessels (Britto-Júnior et al., 2021), and human popliteal artery and vein (Oliveira et al., 2023), 6-ND acts as a potent vasorelaxant mediator. Although the vasorelaxation effect of 6-ND was virtually abolished in vessels where the endothelium has been mechanically removed, the relaxation was not affected by pre-treatment with either L-NAME or the sGC inhibitor ODQ (Lima et al., 2022; Oliveira et al., 2023), indicating a different mechanism of action of the cGMP-dependent NO-mediated vasodilation. Interestingly, the dopamine D2-like receptor antagonist haloperidol (Nyberg et al., 1995) and the selective D2-receptor antagonist L-741,626 (Bowery et al., 1996) also caused endothelium-dependent relaxations of both the human umbilical cord and popliteal vessels (Britto-Júnior et al., 2023; Oliveira et al., 2023), which were insensitive to pre-treatment of the vessels with either L-NAME or ODQ. Since the contractions of human umbilical cord vessels induced by the selective D2-receptor agonist sumanirole (McCall et al., 2005) were blocked by 6-ND pre-incubation (Britto-Júnior et al., 2021), it has been proposed that relaxation induced by 6-ND is due to a highly selective D2-receptor antagonism in the vasculature (Britto-Júnior et al., 2023).

Thus, NO has a dual mechanism of action as a vasorelaxant agent, i.e., stimulation of sCG and synthesis of 6-ND. In marmoset isolated thoracic aortic and pulmonary artery rings, electrical-field stimulation (EFS) caused frequency-dependent contractions, which were significantly increased when the vascular rings were pre-treated with L-NAME, but unaffected by ODQ (Britto-Júnior et al., 2023), indicating that 6-ND could be the major mechanism by which NO causes vasodilation. Similar discrepancies between L-NAME and ODQ were observed in swine carotid, coronary, femoral, and renal arteries (Campos et al., 2024). Since most of the NO-induced vasorelaxation studies were performed in the rabbit aorta, this study investigated whether the rabbit isolated aorta presents the basal release of 6-ND and its role in modulating rabbit aorta contractility.

## Materials and methods

The experiments described in the research protocol (CEUA/UNICAMP: 5538-1/2020; 6287-1/2023) were performed in accordance with the CONCEA and the ARRIVE guidelines. Male New Zealand white rabbits (*Oryctolagus cuniculus*; 2.5–3.0 kg) were acquired from Animais para Pesquisa Criação e Comércio Ltda (Paulínia-SP).

### Rabbit isolated thoracic aorta preparation

The anesthetics xylazine and ketamine (10 and 80 mg/kg, respectively) were injected (IM) after induction by propofol (30 mg/kg; IV). Following exsanguination, the aorta was removed and dissected, and the thoracic aorta was suspended separately in a 5-mL jacketed glass bath containing warmed (37°C) and aerated (95% O_2_/5% CO_2_) Krebs–Henseleit solution (KHS) for 30 min. Some aortas were deliberately denuded from the endothelium by gently eroding them using a stainless-steel forceps.

### Basal release of catecholamines from the rabbit isolated thoracic aorta

The animals were euthanized by exsanguination. Following the removal of the rabbit thoracic aorta, the tissues were suspended in a 5-mL organ bath containing KHS, containing ascorbic acid (3 mM), and continuously gassed with a mixture (95% O_2_/5% CO_2_) at 37°C for 30 min. An aliquot (2 mL) of KHS was collected and stored at −20°C until analysis by liquid chromatography coupled to tandem mass spectrometry (LC-MS/MS; see below). Catecholamine release was also evaluated in endothelium-intact isolated thoracic aortae that were pre-incubated (30 min) with the nitric oxide synthase inhibitor L-NAME (100 μM). When required, the thoracic aorta endothelium was removed by gently rubbing the vessels with forceps.

### Determination of catecholamines by LC-MS/MS

The method used for 6-ND quantification (Campos et al., 2021) was modified to allow the measurement of the four catecholamines in a single chromatographic run. Briefly, the catecholamines from KHS (1 mL) were extracted by solid phase extraction. To 1 mL of KHS was added 50 mL (100 ng/mL) of the deuterated catecholamines used as internal standards, and the samples were homogenized for 10 s. The Strata™-X 33-mm Polymeric Reversed Solid-Phase Extraction (SPE) cartridges were pre-washed with MeOH (1 mL), followed by deionized H_2_O (2 mL). After sample introduction into the cartridge, the cartridge was subsequently washed three times with deionized H_2_O. The catecholamines were then eluted with 900 μL MeOH/H_2_O (90/10, v/v) with formic acid (0.1%). The eluate was evaporated under N_2_ flow at 50°C. The residue was dissolved with 100 μL of acetonitrile/H_2_O (50/50, v/v) with 0.1% formic acid and transferred to vials ready for injection into the mobile phase (75% A composed of deionized H_2_O with 0.1% formic acid (v/v) and 25% B composed of acetonitrile/H_2_O (90/10, v/v) with 0.1% formic acid). The mobile phase perfused an LC ADVp Liquid Chromatograph Shimadzu System coupled to a Shimadzu 8060 triple quadrupole mass spectrometer operating in the ESP^+^ mode at 350 μL/min. The dissolved residues were injected using an SIL-30AC autoinjector, at a temperature of 8°C. The transitions monitored by electrospray multiple reaction monitoring (MRM), injection volume, run-time, and limit of quantitation were described elsewhere (Júnior et al., 2023). The results are expressed as the mean ± standard error of the mean.

### Preparations for isometric tension recordings

The thoracic aortic rings (5-mm length) were fixed vertically between two metal hooks in 10-mL jacketed glass organ bath, containing warmed (37°C) and aerated (95% O_2_ / 5% CO_2_). The upper thread was fixed to an isometric transducer, and the tension measurements were registered in a PowerLab 400TM data acquisition system (Software Chart, version 7.0, ADInstruments, United States). An equilibration period of 60 min was allowed before starting the protocols.

### Evaluation of endothelium integrity

Phenylephrine (1 μM) induced contractions of the endothelium-preserved thoracic aortic rings. After the contraction had reached a plateau, the integrity of the endothelium was assessed by the measurement of the relaxation provoked by acetylcholine (1 μM). A relaxation larger than 50% was indicative of endothelium integrity (Furchgott and Bhadrakom, 1953; Furchgott and Zawadzki, 1980).

### Aorta contractions elicited by electrical-field stimulation

Endothelium-preserved thoracic aorta rings were exposed to EFS at 60 V for 30 s, at 8–32 Hz in square-wave pulses, 0.3 ms pulse width, 0.1-ms delay, using a Grass S88 stimulator (Astro-Medical, United States). The EFS was performed in aortic rings pre-treated (30 min) with L-NAME (100 μM), ODQ (100 μM), methylene blue (10 μM), or indomethacin (3 μM). The EFS was also performed in the presence of 6-ND (1 μM) or L-741,626 (1 μM). A contraction induced by KCl (80 mM) was performed at the start and at the conclusion of the experiment to assess tissue viability.

### Thoracic aorta contractions to dopamine, noradrenaline, and adrenaline

In L-NAME (100 μM)-pre-treated (30 min) endothelium-preserved thoracic aortic rings, concentration–response curves to dopamine (1 nM–300 μM), noradrenaline (1 nM–100 μM), and adrenaline (1 nM–100 μM) were obtained in control rings, and in rings pre-treated with either 6-ND (0.1, 0.3, and 1 μM) or L-741,626 (0.1, 0.3, and 1 μM). The concentration–response curve was kept constant for 15–20 min.

### Thoracic aorta relaxation responses to 6-ND and L-741,626

Endothelium-intact and endothelium-denuded thoracic aortic rings were pre-contracted with endothelin-1 (ET-1, 10 nM). In endothelium-intact rings, after a sustained contraction was obtained, cumulative concentration–response curves to either 6-ND (100 fM–10 nM) or L-741,626 (0.1 pM–10 nM) were obtained in the presence and absence of either L-NAME (100 μM) or ODQ (100 μM). Concentration–response curves to either 6-ND (0.1 pM–10 nM) and L-741,626 (0.1 pM−10 nM) were also obtained in endothelium-denuded rings. In another experiment set, relaxation effects of 6-ND (0.1 pM–10 nM) and acetylcholine (1 nM–100 μM) were evaluated in the presence and absence of methylene blue, an inhibitor of the catalytic function of inducible and constitutive nitric oxide synthesis via the oxidation of enzyme-bound ferrous iron (10 μM, 30 min).

### Chemicals and reagents

Acetylcholine, methylene blue, L-NAME, and phenylephrine were purchased from Sigma-Aldrich Chemicals Co. (St. Louis, Missouri, United States). Adrenaline, dopamine, endothelin-1 (human and porcine), L-741,626, noradrenaline, and 1H-[1,2,4]oxadiazolo [4,3-a]quinoxalin-1-one (ODQ) were purchased from Cayman Chemical (Michigan, United States). 6-Nitrodopamine and 6-nitrodopamine-d_4_ were acquired from Toronto Research Chemicals (Ontario, CA). Adrenaline-d6 hydrochloride, dopamine-d3 hydrochloride, and DL-noradrenaline-d6 hydrochloride were acquired from CDN Isotopes (Pointe-Claire, Canada). Sodium chloride (NaCl), potassium chloride (KCl), calcium chloride (CaCl_2_), magnesium sulfate (MgSO_4)_, sodium bicarbonate (NaHCO_3_), potassium phosphate monobasic (KH_2_PO_4_), and glucose were purchased from Merck KGaA (Darmstadt, Germany). Acetonitrile was obtained from J.T Baker (Phillipsburg, NJ, United States), and formic acid (HPLC grade) was purchased from Mallinckrodt (St. Louis, MO, United States). 6-Nitrodopamine, acetylcholine, adrenaline, dopamine, endothelin-1, L-741,626, L-NAME, methylene blue, noradrenaline, ODQ, and phenylephrine were dissolved in water. ODQ and L-741,626 were diluted initially in distilled water at a concentration of 10 mM, and in the case of ODQ, the solution was warmed at 38°C for 30 min with sonication.

### Data analysis

The EC_50_ values of are presented as the mean ± standard error of the mean (SEM) of *n* experiments. E_max_ values are indicated in mN (“contraction” experiments) or percent levels of relaxations of ET-1-induced precontraction (“relaxation” experiments). Two rings were used, one as control and the other drug-treated. The expression *x/y* indicates the number of experiments, where the number of animals used is represented by *x* and the number of rings by *y*.

The calculation of both pEC_50_ and E_max_, and the statistical tests used, was carried out using GraphPad Prism (GraphPad Software, version 10, San Diego, CA, United States). Nonlinear regression analysis was used to determine the pEC_50_ value with the constraint that F = 0. All concentration–response data were evaluated for a fit to a logistics function in the form E = E_max_/([1 + (10c/10x)n] + F, where E represents the increase in the response contractile induced by the agonist; E_max_ is the effect agonist maximum; c is the logarithm of concentration of the agonist that produces 50% of E_max_; x is the logarithm of the concentration of the drug; the exponential term, n, is a curve fitting parameter that defines the slope of the concentration–response line; and F is the response observed in the absence of the added drug. In addition, standard ANOVA, followed by the Newman–Keuls *post hoc* test, was used when more than two groups were involved. A *p*-value of less than 0.05 was considered statistically significant. In the pharmacological experiments, the number of experiments is expressed as x/y, where x represents the number of aortas (animals) and y the number of rings used in the experiment. One ring was used as the control response, and the other ring was incubated with an antagonist/inhibitor. Student’s two-tailed unpaired *t*-test was used, and the differences between groups with p < 0.05 were considered significant. For E_max_ and pEC_50_ analysis, unpaired Student’s *t*-test was used.

## Results

### Basal release of 6-ND and dopamine

**FIGURE 1:**
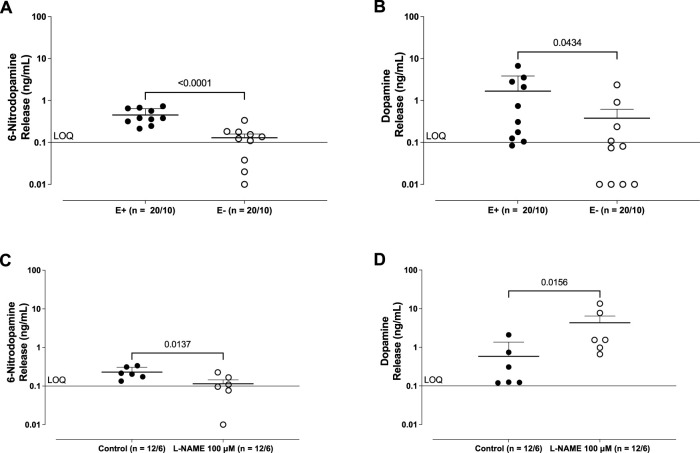
Basal release of 6-nitrodopamine (6-ND; **(A, C)**) and dopamine **(B, D)** from the rabbit isolated aorta. A and B show the effect of endothelium removal (E−) on the basal release of 6-ND and dopamine from the rabbit isolated aorta. **(C, D)** show the effect of pre-incubation (30 min) of L-NAME (100 μM) on the basal release of 6-ND and dopamine from the rabbit isolated aorta. The number of experiments (n) is expressed as x/y, where x represents the number of animals and y the number of rings used. Data are expressed as the mean ± SEM. The unpaired *t*-test was applied in **(A–D)**.

Analysis by LC-MS/MS revealed the basal release of 6-ND (Figures 1A, C) and dopamine (Figures 1B, D) from the thoracic aorta, which was significantly reduced following the mechanical removal of the endothelium. In rings pre-incubated (30 min) with L-NAME (100 μM), 6-ND basal release was significantly decreased (Figure 1C), whereas that of dopamine (Figure 1D) was increased.

Adrenaline basal release was detected only in 3 of 10 aortae (1.2 ± 0.5 ng/mL), and that of noradrenaline was undetectable (LOQ; 0.1 ng/mL) in all samples.

### Effects of L-NAME, indomethacin, ODQ, and methylene blue on EFS-induced aortic contractions

**FIGURE 2:**
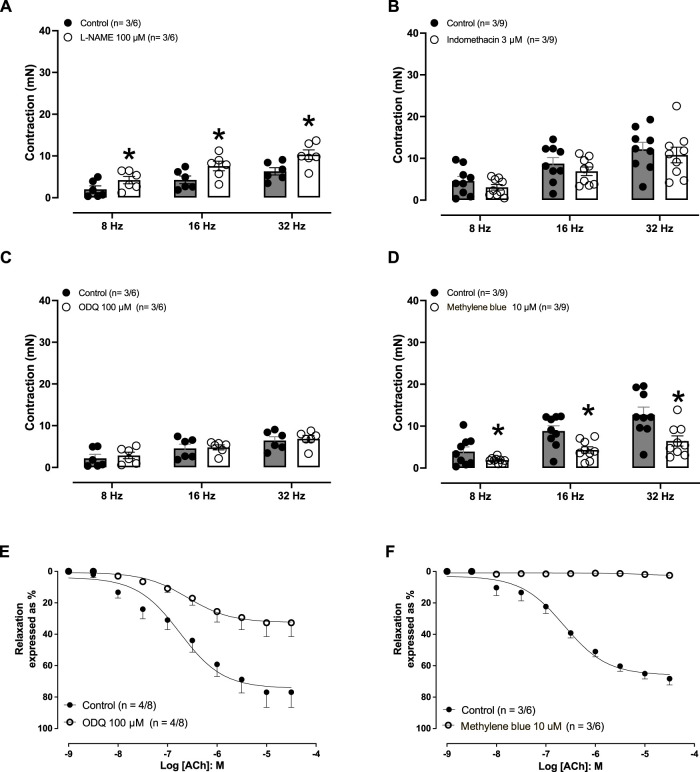
Effect of electric-field stimulation (EFS)-induced rabbit isolated aortic rings. **(A)** Effect of pre-incubation (30 min) of L-NAME (100 μM) on the contractions induced by EFS (8, 16, and 32 Hz) on aortic rings (F = 8.9181; *p* = <0.0001; DF (between groups) = 5 and DFD (within groups) = 30). **(B)** Effect of pre-incubation (30 min) of indomethacin (3 μM) on the contractions induced by EFS on aortic rings (F = 6.7605; *p* = <0.0001; DF (between groups) = 5 and DFD (within groups) = 48). **(C)** Effect of pre-incubation (30 min) of ODQ (100 μM) on the contractions induced by EFS on aortic rings (F = 4.9622; *p* = 0.0019; DF (between groups) = 5 and DFD (within groups) = 30). **(D)** Effect of pre-incubation (30 min) of methylene blue (100 μM) on the contractions induced by EFS on aortic rings (F = 11.5819; *p* = <0.0001; DF (between groups) = 5 and DFD (within groups) = 48). **(E)** Effect of pre-incubation (30 min) of ODQ (100 μM) on the relaxations induced by acetylcholine. **(F)** Effect of pre-incubation (30 min) of methylene blue (10 μM) on the relaxations induced by ACh **(A)** on the aortic rings. The number of experiments (n) in each panel is expressed as x/y, where x represents the number of animals and y the number of rings used. Data are expressed as the mean ± SEM. In **(A–D)**, the statistical analysis was performed by ANOVA (data presented in parenthesis), followed by Student’s unpaired *t*-test (significance illustrated by *). For **(E, F)**, unpaired *t*-test was applied.

Electric-field stimulation (8–32 Hz) produced frequency-dependent contractions in endothelium-preserved aortic rings (Figures 2A–D; *p* = <0.0001), which were augmented when the rings were pre-incubated with L-NAME (100 μM, 30 min; Figure 2A). Pre-treatment of the rings with either indomethacin (3 μM; Figure 2B) or ODQ (100 μM; Figure 2C) had no effect on EFS-induced contractions, whereas pre-treatment with methylene blue (10 μM; Figure 2D) caused significant reductions in the contractions induced by EFS.

In aortic rings with an intact endothelium pre-contracted with endothelin-1 (10 nM), acetylcholine (1 nM–30 μM) induced concentration-dependent relaxations. The rings pre-treated (30 min) with ODQ (100 μM; Figure 2E) or methylene blue (10 μM; Figure 2F) nearly abolished the acetylcholine-induced aortic relaxations (Table 1).

**TABLE 1:** Potency (pEC_50_) and the maximum response (E_max_) of the relaxing effects induced by 6-nitrodopamine (6-ND), L-741,626, or acetylcholine on pre-contracted endothelin 1 (ET-1; 10 nM).

	pEC_50_ (log [M])	E_max_ (%)	n
6-ND			
E+	10.34 ± 0.10	93.01 ± 3.36	5/8
E−	10.12 ± 0.20	41.63 ± 0.19*	5/5
Control	10.33 ± 0.10	93.13 ± 3.39	5/8
L-NAME, 100 μM	10.35 ± 0.19	83.33 ± 5.79	5/8
Control	10.33 ± 0.10	93.13 ± 3.40	5/8
ODQ, 100 μM	10.35 ± 0.18	83.33 ± 5.80	5/8
Control	10.60 ± 0.13	69.85 ± 3.22	3/6
Methylene blue, 10 μM	10.73 ± 0.15	65.58 ± 6.38	3/6
L-741,626			
E+	11.23 ± 0.07	93.64 ± 2.02	5/9
E−	11.01 ± 011	47.26 ± 1.61*	5/9
Control	11.22 ± 0.08	94.34 ± 2.02	5/9
L-NAME, 100 μM	11.41 ± 0.07	89.72 ± 1.76	5/6
Control	11.25 ± 0.07	95.45 ± 5.14	5/9
ODQ, 100 μM	11.18 ± 0.11	97.32 ± 1.72	5/6
Control	11.25 ± 0.08	95.45 ± 5.14	3/8
Methylene blue, 10 μM	11.18 ± 0.11	97.32 ± 1.72	3/7
Acetylcholine			
Control	6.75 ± 0.14	74.40 ± 3.87	4/8
ODQ, 100 μM	6.60 ± 0.24	32.52 ± 3.10*	4/8
Control	6.63 ± 0.09	65.99 ± 2.27	3/6
Methylene blue, 10 μM	—	3.14 ± 4.36*	3/6

pEC_50_ is defined as the negative logarithm of the EC_50_; E_max_ is the maximal effect at high drugs; *p< 0.05 compared with respective control values. Unpaired *t*-test was applied.

### Relaxation of pre-contracted aortic rings by 6-ND and L-741,626: effect of L-NAME, ODQ, and methylene blue

**FIGURE 3:**
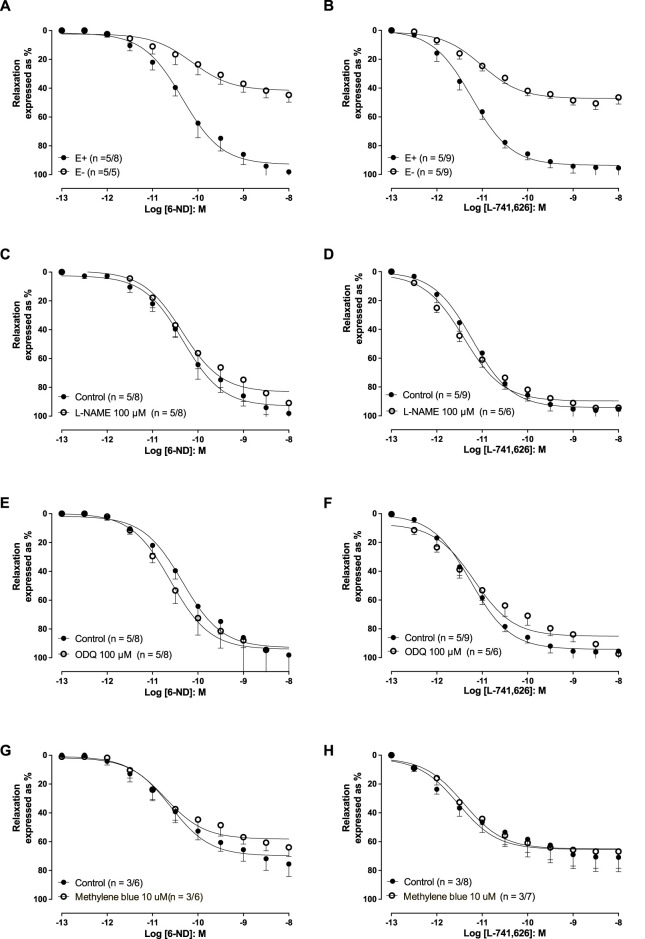
Relaxations induced by 6-ND and L-741,626 in endothelin-1 (10 nM) pre-contracted rabbit isolated aortic rings. **(A, B)** Effect of endothelium removal (E−) on the relaxations induced by 6-nitrodopamine and L-741,626, respectively. **(C, D)** Effect of pre-incubation (30 min) of ODQ (100 μM) on the relaxations induced by 6-ND and L-741,626, respectively. **(E, F)** Effect of pre-incubation (30 min) of methylene blue (10 μM) on the relaxations induced by 6-ND and L-741,626, respectively. Unpaired *t*-test was applied in **(A–H)**.

In endothelin-1 (10 nM)-pre-contracted aortic rings with an intact endothelium, 6-ND (0.1 p.M–10 nM) induced concentration-dependent relaxations, which were markedly reduced by endothelium removal (Figure 3A; Table 1). However, pre-incubation (30 min) with L-NAME (100 μM; Figure 3C), ODQ (100 μM; Figure 3E), or methylene blue (10 μM; Figure 3G) had no significant effect on 6-ND-induced relaxations. The pEC_50_ values for 6-ND did not significantly differ in any group (Table 1).

The addition of L-741,626 (0.1 pM–10 nM) also induced concentration-dependent relaxations in endothelin-1 (10 nM)-pre-contracted aortic rings (Figures 3B, D, F, H; Table 1). In endothelium-denuded rings, the relaxations induced by L-741,626 were significantly reduced (Figure 3B), but pre-incubations with L-NAME (100 μM), ODQ (100 μM), or methylene blue (10 μM) had no effect. The pEC_50_ values for L-741,626 did not significantly differ in any group (Table 1).

### Effect of 6-ND and L-741,626 on dopamine-, noradrenaline-, and adrenaline-induced aortic contractions

**FIGURE 4:**
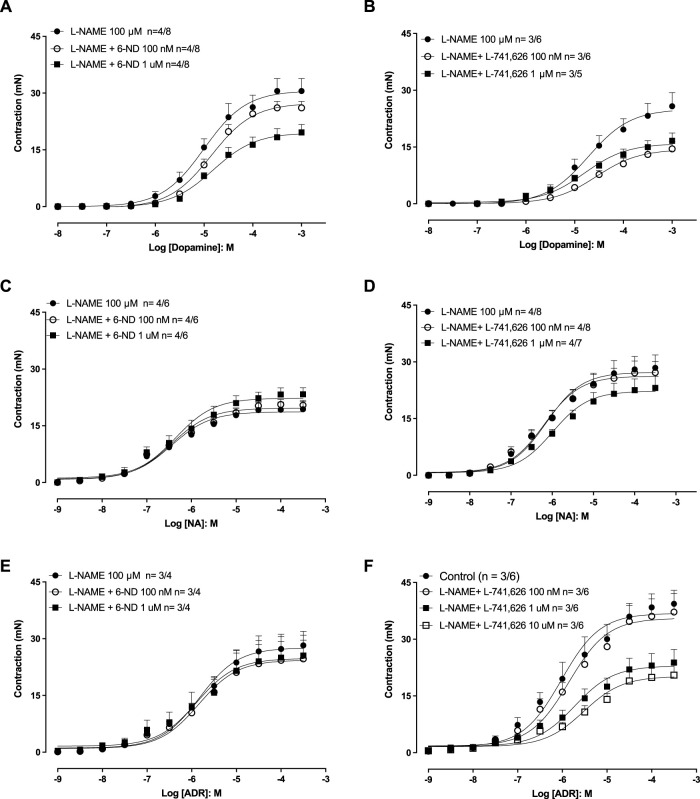
Effects of 6-ND and the selective dopamine D_2_-receptor antagonist L-741,626 on dopamine-, noradrenaline-, and adrenaline-induced aortic contractions. Endothelium-intact aortic rings were all pre-treated with either L-NAME (100 μM, 30 min), after which they were incubated or not with either 6-ND **(A, C, E)** or L-741,626 **(B, D, F)**. Concentration–response curves to dopamine (DA), noradrenaline (NA), and adrenaline (ADR) were then obtained. The number of experiments (n) in each panel is expressed as x/y, where x represents the number of animals and y the number of rings used. Data are expressed as the mean ± SEM. ANOVA, followed by the Newman–Keuls *post hoc* test, was applied in **(A–F)**.

In endothelium-intact aortic rings pre-treated (30 min) with L-NAME (100 µM), the addition of dopamine (Figure 4A), noradrenaline (Figures 4C, D), and adrenaline (Figures 4E, F) induced concentration-dependent contractions. Pre-incubation (30 min) of the aortic rings with 6-ND (0.1 and 1 ⋅ M) significantly reduced the Emax values for dopamine (Figure 4A), whereas the contractile responses to noradrenaline (Figure 4C), and adrenaline (Figure 4E) remained unaffected by 6-ND preincubation (Table 2). 

**TABLE 2:** Potency (pEC_50_) and maximum response (E_max_) of the concentration–response curves to catecholamines in the absence and presence of 6-ND or L-741,626.

Agonist	pEC_50_ (log [M])	E_max_ (mN)	n
Dopamine			
L-NAME, 100 μM	4.99 ± 0.10	30.64 ± 1.30	4/8
L-NAME + 6-ND, 100 nM	4.85 ± 0.05	27.35 ± 0.72*	4/8
L-NAME + 6-ND, 1 μM	4.81 ± 0.09	19.53 ± 0.81*	4/8
L-NAME, 100 μM	4.71 ± 0.12	25.00 ± 1.44	3/6
L-NAME + L-741,626, 100 nM	4.55 ± 0.11	14.36 ± 0.75*	3/6
L-NAME + L-741,626, 1 μM	4.79 ± 0.10	15.91 ± 0.73*	3/5
Noradrenaline			
L-NAME, 100 μM	6.45 ± 0.12	18.67 ± 0.69	4/6
L-NAME + 6-ND, 100 nM	6.44 ± 0.08	19.66 ± 0.49	4/6
L-NAME + 6-ND, 1 μM	6.36 ± 0.11	22.29 ± 0.78	4/6
L-NAME, 100 μM	6.14 ± 0,12	27.24 ± 1.10	4/8
L-NAME + L-741,626, 100 nM	6.19 ± 0.11	26.21 ± 0.99	4/8
L-NAME + L-741,626, 1 μM	5.99 ± 0.12	22.25 ± 0.92*	4/7
Adrenaline			
L-NAME, 100 μM	5.83 ± 0.11	27.59 ± 1.20	3/4
L-NAME + 6-ND, 100 nM	5.84 ± 0.22	24.33 ± 1.98	3/4
L-NAME + 6-ND, 1 μM	5.88 ± 0.18	24.78 ± 1.62	3/4
L-NAME, 100 μM	6.02 ± 012	36.84 ± 1.51	3/6
L-NAME + L-741,626, 100 nM	5.85 ± 0.12	35.53 ± 1.53	3/6
L-NAME + L-741,626, 1 μM	5.72 ± 0.13	22.95 ± 1.14*	3/6
L-NAME + L-741,626, 10 μM	5.51 ± 0.12*	20.18 ± 0.99*	3/6

pEC_50_ is defined as the negative logarithm of the EC_50_; E_max_ is the maximal effect at high drugs; *p< 0.05 compared with respective control values. ANOVA, followed by the Newman–Keuls *post hoc* test, was applied.

In endothelium-intact aortic rings pre-treated (30 min) with L-NAME (100 μM), pre-incubation (30 min) with L-741,626 (100 nM–1 μM) significantly reduced the E_max_ values for dopamine (Figure 4B), noradrenaline (Figure 4D), and adrenaline (Figure 4F; Table 2).

### Effects of 6-ND and L-741,626 on EFS-induced aortic contractions

**FIGURE 5:**
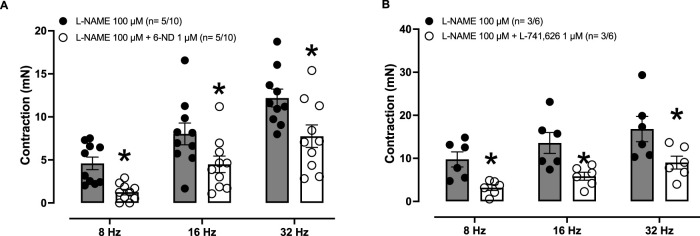
Effects of 6-ND and L-741,626 on EFS-induced contractions in rabbit isolated aortic rings. Endothelium-intact aorta and pulmonary artery rings were pre-treated with L-NAME (100 μM, 30 min), after which they were incubated or not with either 6-ND (1 μM, **panel A**; F = 14.6429; *p* = <0.0001; DF (between groups) = 5 and DFD (within groups) = 54) or the selective dopamine D_2_-receptor antagonist L-741,626 (1 μM, **panel B**; F = 6.4997; *p* = <0.0001; DF (between groups) = 5 and DFD (within groups) = 30). EFS at 8–32 Hz was then applied to tissues. The number of experiments (n) is expressed as x/y, where x represents the number of animals and y the number of rings used. Data are expressed as the mean ± SEM. ANOVA, followed by Student’s unpaired t-test, were applied in (A, B)* Indicates *p*< 0.05 (Student’s unpaired *t*-test).

In endothelium-intact thoracic aortas pre-treated (30 min) with L-NAME (100 μM), applying EFS caused frequency-dependent (8–32 Hz) contractions (Figures 5A, B), which were significantly reduced when the tissues were pre-incubated (30 min) with either 6-ND (1 μM; Figure 5A) or L-741,626 (1 μM; Figure 5B).

## Discussion

The pEC_50_ value of ACh-induced rabbit aorta relaxation varies from 6.74 to 7.30 (Hansen and Nedergaard, 1999; Jayakody et al., 1987; Furchgott and Zawadzki, 1980), similar to the values obtained here (6.75). The pEC_50_ value for 6-ND is 10.34, indicating that 6-ND is the most potent endogenous vasodilator in the rabbit isolated aorta so far described, being over 1,000 times more potent than the classical NO releaser acetylcholine. Indeed, the other endogenous vasodilators also have lower pEC_50_ values as a relaxant of the rabbit isolated aorta, such as bradykinin (8.7; Shikano and Berkowitz, 1987), ATP (4.0; Bény and Brunet, 1988), substance P (6.2; Bény and Brunet, 1988), and CGRP (9.7; Brain et al., 1985). Another important difference between the vasorelaxant effects of these endogenous vasodilators and 6-ND is the dependence of the former on the NO-cGMP pathway, as demonstrated by the inhibition of the acetylcholine-induced relaxations *in vitro* by either the heme-site inhibitor of NO-sensitive soluble guanylate cyclase ODQ (Schrammel et al., 1996) or the unspecific soluble guanylate cyclase inhibitor methylene blue (Mayer et al., 1993). Inhibition of NO synthase by L-NAME also reduces the rabbit aorta-induced relaxations induced by bradykinin (Rees et al., 1990), ATP (Ling et al., 2021), substance P (Najibi, 1997), and CGRP (Al-Zobaidy and Martin, 2014). In contrast, pre-treatment or the vascular tissues with L-NAME or ODQ did not affect the 6-ND-induced relaxations in the rabbit aorta, marmoset thoracic aorta and pulmonary artery rings (Britto-Júnior et al., 2023), human popliteal artery and vein rings (Oliveira et al., 2023), human umbilical artery and vein rings (Britto-Júnior et al., 2021), and *Pantherophis guttatus* (Lima et al., 2022) and *Chelonoidis carbonarius* (Britto-Júnior et al., 2022) aortic rings.

There are two distinct mechanisms by which NO can cause relaxation in the rabbit isolated aorta, namely, the classical NO-sGC-cGMP pathway (Arnold et al., 1977; Murad, 1994) and the 6-ND pathway (Zatz and De Nucci, 2024). Which is more relevant? The results presented here and the abovementioned literature show that the latter is more important. For instance, the contractions induced by EFS were potentiated by NO inhibition; however, in vessels pre-treated with either ODQ or methylene blue, no potentiation was observed. Similar results were observed in both marmoset (Britto-Júnior et al., 2023) and swine arteries (Campos et al., 2024). In addition, the administration of ODQ to rats affected neither the mean arterial blood pressure nor the heart rate, although the *ex vivo* inhibition of soluble guanylate cyclase was achieved (Cechova and Pajewski, 2004). Furthermore, the administration of L-NAME increased blood pressure in both male and female knockout mice for the alpha-1 subunit of soluble guanylate cyclase (sGCa_1_^−/−^ mice; Buys et al., 2008), indicating that the modulatory role of NO in blood pressure is largely independent of soluble guanylate activation.

It is interesting that L-S-nitrosocysteine (L-SNC) has been considered an endothelium-derived relaxing and hyperpolarizing factor (Lewis et al., 2006). Like 6-ND, it relaxes resistance arteries by mechanisms independent of the NO-mediated activation of soluble guanylate cyclase (Woodman et al., 2000; Loyaga-Rendon et al., 2005). S-nitrosothiols induce S-nitrosylation events in different tissues, including endothelial cells and aorta (Stamler et al., 1992; Gow et al., 2002), which could lead to 6-ND formation. Whether L-SNC increases the basal release of 6-ND is currently under investigation.

The endothelium-dependent hyperpolarization (EDH) factor is proposed to be a substance generated by endothelial cells and associated with vascular smooth cell hyperpolarization and relaxation (Feletou and Vanhoutte, 1988). Nitric oxide and NO donors may induce vascular smooth-muscle hyperpolarization (Cohen and Vanhoutte, 1995); however, inhibitors of NO synthase do not inhibit the hyperpolarization induced by the endothelium (Komori et al., 1988), excluding NO as an EDH factor. Endothelium-induced hyperpolarization has also been observed in vascular tissue treated with cyclo-oxygenase inhibitors (Ding and Triggle, 2000), excluding PGE2 and/or prostacyclin as an EDH factor. Although one cannot conclude at the moment that 6-ND is one of the EDH factors, it is definitely the most potent endothelium-derived relaxing factor to be described.

In human isolated umbilical and popliteal vessels (Britto-Júnior et al., 2021; Oliveira et al., 2023), marmoset aorta and pulmonary artery (Britto-Júnior et al., 2023), and swine carotid, coronary, femoral, and renal arteries (Campos et al., 2024), 6-ND promotes *in vitro* endothelium-dependent relaxations via its capacity to selectively antagonize dopaminergic D2-like receptors. Here, using rabbit aortic rings, a similar mechanism of relaxation was found. The selective dopamine D2-receptor antagonist L-741,626 produced concentration-dependent relaxations in a similar fashion to 6-ND, that is, relaxation responses to both agents were greatly reduced by endothelium removal but rather insensitive to L-NAME, ODQ, and methylene blue pre-treatments. It is interesting that in the rabbit isolated aorta, the pEC_50_ value of L-741,626 was 11.23, whereas in the above-mentioned vascular tissues, it varied from 7.47 (swine femoral artery; (Campos et al., 2024)) to 9.31 (human popliteal vein rings; (Oliveira et al., 2023)). This striking difference in potency may be related to a species characteristic since the pEC_50_ value of L-741,626 was also very high (11.15) in the rabbit isolated corpus cavernosum (Lima et al., 2023). Another remarkable difference between these two antagonists is the high selectivity of 6-ND toward the dopaminergic receptor since, in contrast to L-741,626, it had no effect on the contractions of the rabbit aorta induced by either noradrenaline or adrenaline.

Dopamine D2-receptor antagonists, such as the “atypical” antipsychotic clozapine (Ashby and Wang, 1996) and the “classical” antipsychotic haloperidol (Volavka and Cooper, 1987), caused a decrease in both the systolic and diastolic pressure when administered to male healthy volunteers (Pretorius et al., 2001). Overweight people have a higher probability of developing hypertension since there is a linear relationship between weight gain and systolic blood pressure (Kannel et al., 1967; Huang et al., 1998); yet schizophrenic patients treated with clozapine present significant weight gain but without changes in mean arterial blood pressure (Baymiller et al., 2002). Aripiprazole (Hirose and Kikuchi, 2005) or haloperidol administration to healthy volunteers increases cerebral blood flow (Handley et al., 2013). Intravenous administration of haloperidol for acute psychosis treatment (Clinton et al., 1987; Meyer-Massetti et al., 2010; Nassisi et al., 2006) may cause serious hypotension (Yoshida et al., 1993; Harrigan et al., 2004). All the above indicates that dopamine D2-receptor antagonism results in vasodilation; therefore, it is highly probable that 6-ND may have a relevant role in blood pressure regulation *in vivo*. Chronic administration of L-NAME, which leads to a reduction in both NO and 6-ND synthesis, is commonly used as a pre-clinical model of arterial hypertension (Ribeiro et al., 1992). The development of selective dopaminergic D2-receptor antagonists that do not cross the blood–brain barrier may play a relevant therapeutic role in the management of essential hypertension.

The study shows that the basal release of endothelium-derived 6-ND from the rabbit isolated aorta presents some interesting characteristics. It contrasts with other mammalian (human umbilical cord and popliteal vessels and marmoset thoracic aorta and pulmonary artery) and reptilian (tortoise and snake isolated aorta) vascular beds (Zatz and De Nucci, 2024), in that dopamine over 6-ND is the predominant catecholamine released. Indeed, in swine isolated vessels, adrenaline is the major catecholamine released in the isolated carotid artery, dopamine is the major catecholamine released in the isolated renal and femoral arteries, and 6-ND is the major catecholamine released in the isolated coronary artery (Campos et al., 2024). Since they are all endothelium-derived catecholamines, their distinct release pattern may provide a novel mechanism by which the endothelium of each vascular bed controls the respective blood flow.

As observed in the human umbilical cord vessels (Britto-Júnior et al., 2021) and *C. carbonaria* aorta (Britto-Júnior et al., 2022), pre-treatment of the vessels with the dopamine D2 antagonist haloperidol or mechanical removal of the endothelium causes significant reductions in the EFS-induced contractions, reinforcing the concept of endothelium-derived dopamine as a major mediator of vessel contractility (Zatz and De Nucci, 2024). Methylene blue, in contrast to ODQ, caused significant reductions in the EFS-induced contractions, an effect clearly unrelated to soluble guanylate cyclase inhibition. Methylene blue is also known to inhibit cyclo-oxygenase (Evgenov et al., 2002); however, the finding that pre-treatment of the aorta with indomethacin did not affect the EFS-induced contractions indicates that this effect is unrelated to cyclo-oxygenase inhibition. It is interesting that the administration of the dopamine D2-receptor agonist pramipexole decreased the immobility time in the forced swimming test in mice, and this effect was blocked when the animal was pretreated with either haloperidol or methylene blue (Ostadhadi et al., 2016). Whether methylene blue can also act as a dopamine D2-receptor antagonist is currently under investigation.

In conclusion, the results clearly indicate that the balance of endothelium-derived dopamine/6-nitrodopamine synthesis/release may play a pivotal role in the tonus of the vascular smooth muscle and offers novel therapeutic targets for the treatment of arterial hypertension and ischemic vascular diseases.

## Data Availability

The original contributions presented in the study are included in the article/supplementary material; further inquiries can be directed to the corresponding author.
